# Practice variation in urine collection methods among pre-toilet trained children with suspected urinary tract infection: a systematic review

**DOI:** 10.1186/s12887-024-04751-w

**Published:** 2024-05-03

**Authors:** Lucy M Wilson, Clara Tam, Veronica Ka Wai Lai, Motunrayo Ajayi, Mê-Linh Lê, Banke Oketola, Terry P Klassen, Alex Aregbesola

**Affiliations:** 1 Children’s Hospital Research Institute of Manitoba, University of Manitoba, 715 McDermot Ave., Winnipeg, MB R3E 3P4 Canada; 2 Max Rady College of Medicine, University of Manitoba, Winnipeg, Canada; 3 Cumming School of Medicine, University of Calgary, Calgary, Canada; 4 Department of Pediatrics and Child Health, University of Manitoba, Winnipeg, Canada; 5 Neil John Maclean Health Sciences Library, University of Manitoba, Winnipeg, Canada; 6 Centre for Healthcare Innovation, University of Manitoba, Winnipeg, Canada

**Keywords:** Urinary tract infection, Urine collection, Pediatrics, Emergency department, Systematic review

## Abstract

**Background:**

Urinary tract infections (UTIs) are a common cause of acute illness among infants and young children. There are numerous methods for collecting urine in children who are not toilet trained. This review examined practice variation in the urine collection methods for diagnosing UTI in non-toilet-trained children.

**Methods:**

A systematic review was completed by searching MEDLINE (Ovid), Embase (Ovid), CENTRAL (Ovid), PsycInfo (Ovid), CINAHL (EBSCO), and JBI (Ovid) from January 1, 2000 until October 9, 2021 and updated on May 24, 2023. Studies were included if they were conducted in an acute care facility, examined pre-toilet trained children, and compared one urine collection method with another for relevant health care outcomes (such as length of stay in an ED, or re-visits or readmissions to the ED) or provider satisfaction. Two independent reviewers screened the identified articles independently, and those included in the final analysis were assessed for quality and bias using the Newcastle-Ottawa Scale.

**Results:**

Overall, 2535 articles were reviewed and 8 studies with a total of 728 children were included in the final analysis. Seven studies investigated the primary outcome of interest, practice variation in urine collection methods to diagnose a UTI. The seven studies that investigated novel methods of urine collection concluded that there were improved health care outcomes compared to conventional methods. Novel methods include emerging methods that are not captured yet captured in clinical practice guidelines including the use of ultrasound guidance to aid existing techniques. Three studies which investigated healthcare provider satisfaction found preference to novel methods of urine collection.

**Conclusions:**

There is significant practice variation in the urine collection methods within and between countries. Further research is needed to better examine practice variation among clinicians and adherence to national organizations and societies guidelines. PROSPERO registration number CRD42021267754.

**Supplementary Information:**

The online version contains supplementary material available at 10.1186/s12887-024-04751-w.

## Background

Urinary tract infections (UTIs) are a common cause of acute illness among infants and young children, with an estimated prevalence of around 7% in children under two years of age [1]. Many of these children present to the Emergency Department (ED) acutely unwell with an unexplained fever and non-specific clinical symptoms.

In cases of unexplained fever in a young child a diagnostic workup will include assessing for a UTI. To diagnose a UTI, a urine specimen must be collected from the child [2]. For children who are not toilet trained there is a high risk of contamination [3]. Thus, it is important to choose an appropriate urine collection method. Possible urine collection methods include bagged urine sample, clean catch specimens, pad sampling, catheterization, and suprapubic aspiration [4]. Catheterization and suprapubic aspiration are invasive methods but yield the lowest contamination rates. Bagged urine collection and clean catch specimens and pad sampling are non-invasive and thus are less painful for children but have higher rates of contamination [4]. Recently, there has been the emergence of new techniques aimed to improve urine collection effectiveness and patient experience. Methods include the Quick-Wee method, in which gauze soaked in cold saline is placed over the suprapubic area to stimulate voiding, and the bladder stimulation technique which involves gently tapping over the suprapubic area, followed by a lumbar massage, and repeating these manoeuvres until the child voids [5, 6]. An emerging invasive technique is adding point-of-care ultrasound to visualize the bladder before attempting catheterization [7]. 

Given the numerous methods available to collect a urine sample, it is speculated that there is a wide variation among emergency physicians about the appropriate urine collection method. This variation is also found in the recommendations made by various national health organizations and societies [2, 8]. This study aims to examine the presence of practice variation in the urine collection methods for diagnosing UTI in non-toilet-trained children and its effects on healthcare outcomes and utilization. The secondary objectives are [1] to characterize practice variation in urine collection methods among health care providers and [2] to determine practice compliance and healthcare providers’ satisfaction of urine collection methods with the local clinical practice guidelines.

## Methods

The systematic review was reported in accordance with the PRISMA 2020 statement [9], and the protocol was registered with PROSPERO in August 2021 (registration number CRD42021267754).

### Search strategy and study selection

A systematic search of the literature was completed to identify potentially relevant studies. An experienced health sciences librarian (M.L.) designed and executed the search strategy, using a combination of subject terms and keywords that were later translated for each database. The MEDLINE search was peer-reviewed by an independent health sciences librarian as per the PRESS guidelines [10]. Searches were performed in MEDLINE (Ovid), Embase (Ovid), CENTRAL (Ovid), PsycInfo (Ovid), CINAHL (EBSCO), JBI (Ovid), and Google Scholar from January 1, 2000 until October 9, 2021 and an updated search strategy was performed on May 24, 2023. The search was limited to 2000 onwards to ensure that any results or findings were relevant to current guidelines. The searches were designed to be broad, and no restrictions were used. Identified studies were first deduplicated using the Ovid de-duplication and then were deduplicated in EndNote X20 before being uploaded to Covidence. Clinical trial registry searches were conducted in ClinicalTrials.gov and the WHO International Clinical Trials Registry Platform for any ongoing or upcoming trials. Our Ovid multi-file search strategy is available in Appendix A and all search strategies are available at 10.34990/FK2/IZ3M32.

We included studies if they met all of the following criteria: (1) conducted in an acute care facility that care for children, including EDs or Urgent Care Centres, (2) used at least one urine collection method to diagnose UTI, (3) compared one urine collection method with another urine collection method, (4) included one of the relevant outcomes, such as, healthcare outcomes (including length of stay in an ED, re-visits or readmissions to the ED), or healthcare utilization (such as ambulance transfers, interfacility/inter-ED transfers, potentially avoidable transfers), (5) observational studies, including cohort and cross-sectional studies, or controlled-clinical study, and (6) were published in the English language. Studies were excluded if the outcome was not relevant, did not include pre-toilet trained patients (defined as age ≤ 3 years), or if the setting was outside of an acute care facility.

The articles identified in the literature search were first screened by title and abstracts using Covidence systematic review software for inclusion in the systematic review by independent reviewers (LMW, CT, MA, VKWL, BO) [11]. The independent reviewers then reviewed the full-length manuscripts for inclusion in the final analysis. Disagreements during screening were resolved by discussion between reviewers or in consultation with a third reviewer (AA).

### Data extraction

The data from the included studies was extracted by two independent reviewers (LMW, CT, BO). Reviewers used a customized data extraction tool to identify key characteristics of the articles, including information on study design, objectives, population, intervention, outcomes, and conclusion details. The tool was used to pilot test five studies after which it was adopted for the entire included studies. A third reviewer (AA) examined the data to ensure accuracy and identify any errors when appropriate.

### Risk of bias assessment

The included articles were assessed for quality and bias using the Newcastle-Ottawa Scale (NOS) [12], a validated critical appraisal checklist for nonrandomised observational studies. A modified version of the NOS for cohort studies was used to assess the cross-sectional studies. We substituted the term “cohort” with “sample” in the selection domain. We removed questions 2 and 3 concerning follow-up and introduced a question that evaluates the statistical tests conducted in the outcome domain. The NOS rates articles on a star system in order to evaluate the selection of study groups, comparability of groups, and ascertainment of exposure or outcome of interest [12]. Two reviewers (LMW, CT, BO) independently completed the risk of bias assessment, and disagreements were resolved by a third reviewer (AA).

### Data analysis and synthesis

Data were collected and managed using Excel and Covidence [11, 13]. Individual article characteristics were summarized and presented in tabular form, and the results were compared based on the primary and secondary objectives.

## Results

### Search results

**Fig. 1:**
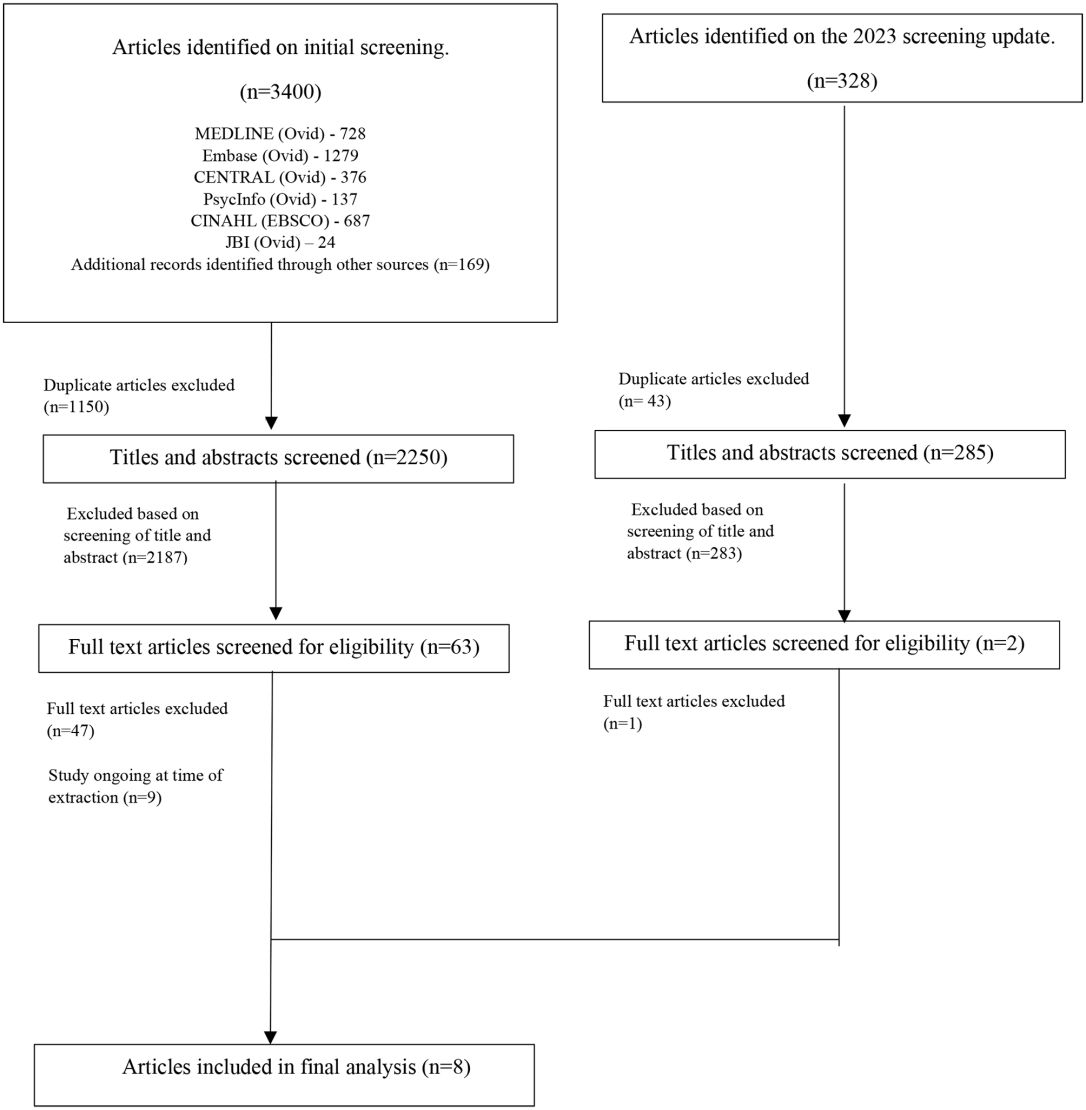
PRISMA flow diagrams of articles identified on initial screening, and updated in October 2022 and included in the final analysis

The search and study screening were conducted initially in October 2021. The initial systematic search of the databases identified 3400 articles, and the updated search identified an additional 328 articles. After duplicates articles were excluded, 2535 titles and abstracts were reviewed, 65 full text articles screened, and 8 studies were included in the final analysis. Full details are presented in Fig. 1, the PRISMA diagram.

### Characteristics of included studies

Characteristics of studies included in the analysis are presented in Table 1. Of the 8 included studies, 2 were conducted in the United States [6, 14], 1 in Turkey [7], 1 in Israel [15], and 4 in Australia [5, 16–18]. All studies were completed in EDs. The American studies by Baumann et al., and Ravichandran et al. compared ultrasound guided catheterization [14], and bladder stimulation technique to conventional catheterization [6, 7], respectively. The study by Akca Caglar et al. was conducted in Turkey and compared point-of-care ultrasound-guided catheterization to conventional catheterization [7]. Kozer et al. investigated transurethral catheterization to suprapubic aspiration in Israel [15]. Finally, the Australian studies by Ho et al., Kaufmann et al. (2017), Kaufman et al. (2019) and Lennon et al. compared a variety of urine collection methods. Ho et al., compared urine collection pads to clean catch urine specimens [16]. Kaufman et al. (2017) compared the Quick-wee method to clean catch urine specimens [5], whereas Kaufman et al. (2019) compared the Quick-wee method to clean catch urine, urine collection bags, catheterization, and suprapubic aspiration [17]. Lennon et al. compared point-of-care ultrasound to assess bladder volume and stimulate the micturition reflex to traditional clean catch urine collection [18]. 

**Table 1:** Study characteristics

First author, year	Country, Continent	Study design	Study period	Type of centre	Study objective	Urine collection method preferred/concluded	Number of participants	Proportion male, %	Mean age (SD) / Median age [IQR]; age range, months	Funding Type
Akca Caglar et al., 2021	Turkey, Europe	Prospective cohort	December 2019 to January 2020	ED	To compare success rates of bladder catheterization in patients with and without POCUS to guide the timing of the procedure. To use POCUS during bladder catheterization in children to evaluate its efficacy in this invasive procedure.	Point-of-care ultrasonography guided catheterization	110	39.1	5.5 (5.5); 0.4 to 24	N/A
Baumann et al., 2007	United States, North America	Randomised controlled trial	July 2005 to June 2006	ED	To assess caregiver and health care provider satisfaction using bladder catheterization and catheterization aided by volumetric bladder ultrasonography.	Ultrasound guided catheterization	93	NR	NR; < 36	N/A
Ho et al., 2013	Australia, Oceania	Prospective cohort	NR	ED	To compare the contamination rate in UCP samples with CCU. To compare the time taken for UCP collection and CCU. Undertake a comparative cost analysis of the two urine collection techniques. To survey parents/carers perceptions of the two urine techniques.	Urine collection pad	22	50.0	13.7; < 36	N/A
Kaufman et al., 2017	Australia, Oceania	Randomised controlled trial	September 2015 to April 2016	ED	To determine if a simple stimulation method increases the rate of infant voiding for clean catch urine within five minutes.	Quick-wee stimulation with clean catch urine	354	50.0	5.4 (3.1); 0 to 1	Government; Academic of research institution; Private
Kaufman et al., 2019	Australia, Oceania	Case cohort	NR	ED	To determine the cost-effectiveness of urine collection methods for precontinent children.	Catheterization / Voiding simulation (Quick-wee)	NR	NR	NR; 0 to 24	Government; Academic of research institution
Kozer et al., 2006	Israel, Asia	Randomised controlled trial	April 2004 to April 2005	ED	To compare in infants who are younger than 2 months the severity of pain during SPA with the pain during TUC	Transurethral catheterization	51	60.8	1.0 (0.4); 0 to 2	Academic of research institution
Lennon et al., 2023	Australia, Oceania	Randomised controlled trial	January 2017 to December 2019	ED	To show a reduction in the time taken to collect a CCU sample in ultrasound assisted collection compared to standard CCU. To show a reduced number of urine collection attempts in the ultrasound group compared to CCU group.	Ultrasound facilitated clean catch urine	73	60.0	10.4 / 9.8; 0 to 36	N/A
Ravichandran et al., 2021	United States, North America	Prospective cohort	September 2017 to May 2018	ED	To evaluate the acceptability and feasibility of incorporating the bladder stimulation technique for CCU collection into routine clinical practice in a busy, urban, academic pediatric emergency department.	Bladder stimulation test with clean catch urine	124	50.0	3.3 [2.9]; < 6	Government; Academic of research institution; Private

*Note* ED = Emergency Department, CCU = clean catch urine, POCUS = point-of-care ultrasound, UCP = urine collection pad, SD = Standard Deviations, IQR = Interquartile Range, NR = Not Reported, N/A = Not Applicable

Overall, five studies investigated the use of catheterization for urine collection as compared to other urine collection methods. Two studies sought to explore the role of ultrasound-guided catheterization as a means of urine collection method. Four studies sought to investigate novel non-invasive urine collection methods as compared to conventional techniques. Of these studies, three focused solely on non-invasive collection methods, and they were all conducted in Australia.

### Outcomes

#### Primary outcome: practice variation and healthcare outcomes and utilization

Eight studies were included in the final analysis. Of the studies that examined health care outcomes and utilization, no studies looked at outcomes of interest as defined in the methods section.

In total all eight studies investigated the outcome of interest of practice variation in urine collection method [5–7, 14–17]. Full details are presented in Table 2. Every included study adhered to the local clinical guidelines cited in the paper. Of these studies, five cited the American Academy of Pediatrics [2] as the clinical practice guidelines, which recommends invasive techniques including bladder catheterization or suprapubic aspiration as first line collection method [2]. Two studies referenced the UK NICE Guidelines [8], which recommends clean catch urine and non-invasive methods as first line collection method [8]. One study referenced American and British recommendations that were not the American Academy of Pediatrics, or the UK NICE Guidelines, respectively [19, 20]. They also cited Australian clinical practice guidelines [21, 22]. The final study did not report a clinical practice guideline but was conducted in the United Kingdom. The study listed clean catch urine as their recommended method which adheres to the UK NICE guidelines.

**Table 2:** Included studies and the clinical practice guidelines on urine collection methods

First author, year	Clinical practice guideline	Country of clinical practice guideline	Year of clinical practice guideline	Strong / first line recommended urine collection method from local clinical guideline	Adherence to local clinical guidelines	Author’s recommended urine collection method
Akca Caglar et al., 2021	American Academy of Pediatrics	United States	2011	Bladder catheterization / suprapubic aspiration	Yes	Point-of-care ultrasonography guided catheterization
Baumann et al., 2007	American Academy of Pediatrics	United States	NR	Bladder catheterization / suprapubic aspiration	Yes	Ultrasound guided catheterization
Ho et al., 2013	NR	United Kingdom	NR	Clean catch urine	Yes	Urine collection pad
Kaufman et al., 2017	UK NICE Guidelines	United Kingdom	2007	Clean catch urine	Yes	Quick-wee stimulation with clean catch urine
Kaufman et al., 2019	UK NICE Guidelines; American Academy of Pediatrics	United Kingdom / United States	2007 / 2011	Clean catch urine; Bladder catheterization / suprapubic aspiration	Yes	Catheterization / Voiding simulation (Quick-wee)
Kozer et al., 2006	American Academy of Pediatrics	United States	1999	Bladder catheterization / suprapubic aspiration	Yes	Transurethral catheterization
Lennon et al., 2023	KHA-CARI guideline; New South Wales Guidelines; Agency for Healthcare Research and Quality; National Collaborating Centre for Women’s and Children’s Health (UK)	Australia / Australia / United Kingdom / United States	2015/2005/2009/2007	Clean catch urine; Clean catch urine; Bladder catheterization / suprapubic aspiration	Yes	Ultrasound facilitated clean catch urine
Ravichandran et al., 2021	American Academy of Pediatrics	United States	2011	Bladder catheterization / suprapubic aspiration	Yes	Bladder stimulation test with clean catch urine

*Note* NR = Not Reported, N/A = Not Applicable

Seven studies included the primary outcome of interest, practice variation in urine collection methods and its effects on healthcare outcomes and utilization [5–7, 15–18]. Table 3 incudes the outcomes of interest. The study by Akca Calgar et al. compared the success rate of point-of-care ultrasound-guided catheterization versus conventional catheterization. The study found a statistically significant difference (*p* = 0.03) between the success rates of ultrasound-guided catheterization (93%) and conventional catheterization (78%) [7]. The study did not find a significant difference between success rates stratified by patient sex.

**Table 3:** Primary outcome: healthcare outcomes and utilization

First author, year	Intervention vs. Comparator	Comparator	Primary outcome	Primary outcome effect estimate	Intervention, N	Comparator, N	Primary outcome results (unadjusted)	Variables used to adjust Primary outcome	Primary outcome results [adjusted]	Conclusion
Invasive Methods										
Akca Caglar et al., 2021	Point-of-care ultrasonography guided catheterization	Conventional catheterization	Success rate	Percentage	54	56	Intervention = 78.0; Comparator = 93.0; *p* = 0.03	N/A	N/A	Use of point-of-care ultrasonography (POCUS) during bladder catheterization in children was found to be effective and successful. The detection of any amount of urine in the bladder using POCUS increases the success rate of bladder catheterization.
Kozer et al., 2006	Transurethral catheterization	Suprapubic aspiration	Neonatal acute pain scale (DAN); Pain visual analog scale by a nurse; Duration of cry (seconds)	Mean difference	27	24	2.5, 95% CI (1.4 to 3.7); 19.6, 95% CI (7.4 to 31.8); 13.2, 95% CI (-4.3 to 30.7)	Age, use of analgesics	NR	In infants younger than 2 months, suprapubic aspiration is more painful than transurethral catheterization.
Mixed invasive and non-invasive methods										
Kaufman et al., 2019	Urine bag vs. Clean catch	Clean catch (CC) vs. Quick-wee (Q-W) vs. Catheter (C) vs. Suprapubic aspirate (SPA)	Time; Success; Average cost-effectiveness per successful collection	Mean (SD); Percentage; Cost	Time, n; Success, n Urine bag 23; 169	Time, n; Success, n CC = 218; 218 Q-W = N/A; 174 C = 45; 148 SPA 20; 38	Urine bag = 85 (67) min; 96 (48); £92.60 CC = 31 (42) min; 64 (45); £52.84 Q-W = 5 (N/A) min; 30 (47); £41.32 C = 12 [7] min; 90 (45); £25.98 SPA = 8 [4] min; 44 [22]; £37.80	N/A	N/A	Catheterization is the most cost-effective urine collection method, and quick-wee is the most cost-effective non-invasive method. Urine bags are the most expensive.
Ravichandran et al., 2021	Bladder stimulation for clean catch urine	Catheterization	Voiding within 300 s; Training was effective; Procedure was easy to perform; Patient tolerated procedure well with minimal discomfort	Median [IQR]; Percentage	47	59	Intervention 73.0 [125], *p* < 0.001; 98.0%, *p* < 0.001; 98.0%, *p* < 0.001 Comparator 9.5 [17], *p* < 0.001; 2.0%, *p* < 0.001; 10.0%, *p* < 0.001	Age, sex, adequate fluid intake, route/method of fluid intake, voiding in the hour preceding BST, and provider experience in performing the BST	NR	The bladder stimulation technique for clean catch urine collection is a well-tolerated and well-received approach that can easily be implemented into clinical practice with minimal training.
Non-invasive Methods										
Ho et al., 2013	Urine collection pad	Clean catch urine	Time to urine collection	Median [IQR]	22	22	Intervention = 30 [10-1135]; Comparator = 107.5 [30–330]; *p* < 0.002	N/A	N/A	This study suggests that urine collection pads are practicable in Australasian EDs and may lead to faster diagnosis, disposition and reduced hospital stay.
Kaufman et al., 2017	Quick-wee	Clean catch urine	Voided < 5 min; Voided and successful catch	Mean difference	174	170	19.0, 95% CI (11 to 28), *p* < 0.001; 21.0, 95% CI (13 to 29), *p* < 0.001	Age, sex	NR	Quick-wee is a simple cutaneous stimulation method that significantly increases the five-minute voiding and success rate of clean catch urine collection.
Lennon et al., 2023	Ultrasound facilitated clean catch urine	Conventional clean catch urine	To show a reduction in the time taken to collect a CCU sample in ultrasound assisted collection compared to standard CCU	Mean (SD); Median [IQR]	37	36	Intervention = 82 (90) min, 55 [81] min; Comparator = 52 (42) min, 40 [52] min; *p* = 0.038	N/A	NR	Use of bladder ultrasound to facilitate clean-catch urine collection significantly improved times to collection by approximately 15 min.

*Note* ED = Emergency Department, N/A = Not Applicable, NR = Not Reported, IQR = Interquartile Range, 95% CI = 95% Confidence Interval, SD = Standard Deviation

Four studies compared clean catch urine samples to alternative urine collection methods. Ho et al. compared the time needed to collect a urine sample between the clean catch urine method to the pad sampling technique. The study found that the pad sampling was statistically significantly faster than clean catch urine collection (30 min [10-1135] vs. 107.5 min [30–330]; *p* < 0.002) [16]. 

Kaufman et al. (2017) investigated the voiding time and success rate of clean catch urine sampling to the Quick-Wee method. The study found that the Quick-Wee method decreased the five-minute voiding time (mean difference 19.0, 95% CI 11–28) and increased the success rate of urine collection (mean difference 21.0, 95% CI 13–29) [5]. It adjusted for age and sex.

Kaufman et al. (2019) compared clean catch urine collection to four other methods – urine bags, the Quick-Wee method, catheterization, and suprapubic aspiration. The study investigated the cost-effectiveness of urine collection methods and measured the time to collect a urine sample, and the success rate of each method as part of its cost-effectiveness study. Catheterization was found to be the most cost-effective (£25.98), followed by suprapubic aspiration (£37.80), voiding simulation (£41.32), clean catch (£52.84), and urine bag (£92.60). Its model estimated that the Quick-Wee was the quickest voiding time (5 min), followed by suprapubic aspiration (8 min ± 4 min), catheterization (12 min ± 7 min), clean catch (31 min ± 42 min), and finally urine bag (85 min ± 67 min). The highest success rate was urine bag (96% ± 48%), followed by catheterization (90% ± 47%), clean catch urine (64% ± 45%), suprapubic aspiration (44% ± 22%), and finally Quick-Wee (30% ± 47%) [17]. 

Lennon et al. examined the time to obtain a clean catch urine sample. The study compared using point-of-care ultrasound to measure bladder volume and stimulate the micturition reflex prior to initiating urine collection to standard clean catch urine collection methods. The study found that ultrasound assistance had a statistically significant reduction in the mean (52 min ± 42 min) and median (40 min, IQR 52 min) time to collection compared to standard clean catch urine practices (mean 82 min ± 90 min; median 55 min, IQR 81 min; *p* = 0.038) [18]. 

Kozer et al. measured the difference in neonatal pain and duration of cry between transurethral catheterization and suprapubic aspiration. Pain was rated using two independent measures, one by parents and nurses, the other by investigators on the research team. Suprapubic aspiration was found to be more painful (mean difference 2.5, 95% CI 1.4–3.7; 19.6 95% CI 7.4–31.8) and have a longer duration of cry than catheterization (13.2 Sect. 95% CI -4.3 to 30.7 s) [15]. The study adjusted for age and use of analgesia.

The final study by Ravichandran et al. compared the voiding time within 300 s between bladder stimulation to conventional catheterization. There was a statistically significant difference between bladder stimulation (median 73 s, IQR 125 s), with 38% success rate and catheterization (median 9.5 s, IQR 17) with success rate (77%). The study adjusted for a number of factors [6]. 

#### Secondary outcome: practice variation in urine collection method

The review did not identify any papers that assessed physician compliance to the local guidelines. However, we used authors recommendations on urine collection method highlighted in Table 2 as an indirect marker of physician adherence. All eight studies included this marker, and this was discussed as part of the primary outcome of interest.

#### Secondary outcome: healthcare professional satisfaction

Three studies were included in the final secondary outcome, practice compliance and satisfaction of healthcare provider with various urine collection methods (Table 4) [5, 6, 14]. Baumann et al. investigated caregiver, nurse, and physician satisfaction using standardized, seven-point Likert scale questionnaires comparing ultrasound-guided catheterization and conventional catheterization. The study found that both caregivers and healthcare providers had greater satisfaction with ultrasound-guided catheterization (nurses: 3.0, 95% CI 2.5–3.5; physicians: 4.3, 95% CI 3.7–4.9) compared to conventional catheterization (nurses: 5.5, 95% CI 5.1–6.0; physicians: 5.7, 95% CI 5.2–6.1) and would prefer this modality with future urine collection attempts [14]. 

**Table 4:** Secondary outcome: healthcare professional satisfaction

First author, year	Intervention vs. Comparator	Secondary outcome	Secondary outcome effect estimate	Intervention, N	Comparator, N	Secondary outcome results (unadjusted)	Variables used to adjust Secondary outcome	Secondary outcome results [adjusted]	Conclusion
Baumann et al., 2007	Ultrasound guided catheterization vs. Conventional catheterization	Caregiver satisfaction; Nurse satisfaction; Physicians’ satisfaction	Number	45	48	Intervention 4.5, 95% CI (3.9 to 5.2), *p* < 0.0001; 3.0, 95% CI (2.5 to 3.5), *p* < 0.0001; 4.3, 95% CI (3.7 to 4.9), *p* < 0.0001 Comparator 6.4, 95% CI (6.1 to 6.8), *p* < 0.0001; 5.5, 95% CI (5.1 to 6.0), *p* < 0.0001; 5.7, 95% CI (5.2 to 6.1), *p* < 0.0001	N/A	N/A	Both caregivers and health care providers expressed greater satisfaction with ultrasound and were more likely to prefer this imaging modality with future catheterization attempts.
Kaufman et al., 2017	Quick-wee vs. Clean catch urine	Clinical satisfaction	Mean difference	174	170	1.0, 95% CI (0.6 to 1.4)	Age, sex	NR	Quick-wee is a simple cutaneous stimulation method that significantly increases the five-minute voiding and success rate of clean catch urine collection.
Ravichandran et al., 2021	Bladder stimulation for clean catch urine vs. Catheterization	Provider satisfaction	Percentage	47	59	Intervention = 98.0%; Comparator = 54.0%; *p* < 0.001	Age, sex, adequate fluid intake, route/method of fluid intake, voiding in the hour preceding BST, and provider experience in performing the BST	NR	The bladder stimulation technique for clean catch urine collection is a well-tolerated and well-received approach that can easily be implemented into clinical practice with minimal training.

*Note* ED = Emergency Department, N/A = Not Applicable, NR = Not Reported, IQR = Interquartile Range, 95% CI = 95% Confidence Interval, SD = Standard Deviation

Kaufman et al. (2017) looked at parental and clinical satisfaction between the Quick-Wee method and clean catch urine using a five-point Likert scale survey. The Quick-Wee method was preferred over clean catch urine by both parents and clinicians (mean difference 1.0, 95% CI 0.6–1.4) [5]. 

The final study by Ravichandran et al. investigated provider satisfaction between the bladder stimulation method and catheterization. The study used a five-point Likert scale to elicit provider’s perspectives on the two collection methods. It found that clinicians felt that compared to catheterization, the bladder stimulation technique was effective (*p* < 0.001), easy to perform (*p* < 0.001), well tolerated by patients (*p* < 0.001), and had high parental satisfaction (*p* = 0.002) [6]. 

### Risk of bias across studies

The NOS [12] was used to evaluate the included studies. The results of the assessment are presented in Table 5. All 8 studies were rated as having a low risk of bias in the areas of representativeness of exposed sample, definition of controls, method of ascertainment for cases and controls, comparability bias, and follow-up bias. Four of the eight studies were deemed high risk for selection bias relating to the ascertainment of the exposure. One study was deemed high risk for its non-response rate. Finally, two studies were evaluated as high risk for assessment bias.

**Table 5:** Secondary outcome: healthcare professional satisfaction

First author, year	Study design	Case definition adequate?	Representativeness of the exposed sample / cases (selection bias)	Selection of the non exposed sample / controls (selection bias)	Definition of controls	Ascertainment of exposure (selection bias)	Same method of ascertainment for cases and controls	Demonstration that outcome of interest was not present at start of study (selection bias)	Comparability of samples on the basis of the design or analysis (comparability bias)	Non-Response rate	Assessment of outcome (assessment bias)	Was follow up long enough for outcomes to occur (follow-up bias)	Adequacy of follow up of cohorts (follow-up bias)
Akca Caglar et al., 2021	Prospective cohort	N/A	Low risk	Low risk	N/A	Low risk	N/A	Low risk	Low risk	N/A	Low risk	Low risk	Low risk
Baumann et al., 2007	Randomised controlled trial	Low risk	Low risk	Low risk	Low risk	High risk	Low risk	N/A	Low risk	Low risk	N/A	N/A	N/A
Ho et al., 2013	Prospective cohort	N/A	Low risk	Low risk	N/A	Low risk	N/A	Low risk	Low risk	N/A	High risk	Low risk	Low risk
Kaufman et al., 2017	Randomised controlled trial	Low risk	Low risk	Low risk	Low risk	High risk	Low risk	N/A	Low risk	Low risk	N/A	N/A	N/A
Kaufman et al., 2019	Case Cohort	Low risk	Low risk	Low risk	N/A	Low risk	N/A	Low risk	Low risk	N/A	Low risk	Low risk	Low risk
Kozer et al., 2006	Randomised controlled trial	Low risk	Low risk	Low risk	Low risk	High risk	Low risk	N/A	Low risk	High risk	N/A	N/A	N/A
Lennon et al., 2023	Randomised controlled trial	Low risk	Low risk	Low risk	Low risk	High risk	Low risk	N/A	Low risk	Low risk	N/A	N/A	N/A
Ravichandran et al., 2021	Prospective cohort	N/A	Low risk	Low risk	N/A	Low risk	N/A	Low risk	Low risk	N/A	High risk	Low risk	Low risk

*Note* N/A = Not Applicable

## Discussion

The results of this systematic review have shown that there was variation in the practice of urine collection methods within and between countries. When examining the adherence to the recommendations of national pediatric associations and societies all eight studies adhered to the guidelines cited, however, only Baumann et al., Lennon et al., and Ravichandran et al. cited the recommended guidelines of the country the study was conducted in [6, 14, 18]. Of those that referenced guidelines from other countries, only two acknowledged the preferred collection method of their home country. Ho et al. stated that a recent Australasian study found that clean catch urine was the preferred method across 13 study sites, which is in keeping with the conclusions in their paper [16, 23]. Lennon et al. cited two Australian clinical practice guidelines. The first was by Kidney Health Australia, which recommends clean catch urine, midstream urine, or catheterization as standard urine collection methods [22]. The other reference was a 2005, now rescinded, policy from the New South Wales government in Australia, which recommended clean catch urine samples in cases of children presenting with fevers of unknown origin [21]. The remaining four studies did not reference their own national guidelines, but instead referenced the guideline that fits their research question.

While all eight studies used methods in their studies that adhered to the local guidelines, the authors conclusions regarding a recommended collection method did not match the current guidelines in seven of the studies. The recommendation suggested by Kozer et al. to use catheterization as the preferred urine collection methods aligned with the American Academy of Pediatrics [15]. Of the remaining seven studies, six of them aligned with the clinical practice guidelines on whether invasive or non-invasive methods were preferred. However, the recommended means of urine collection was different than what their society recommended. The remaining study, by Ravichanran et al. concluded that bladder stimulation technique is preferred over suprapubic aspiration in infants admitted to the NICU, which differs from the American Academy of Pediatrics recommendation of performing catheterization or suprapubic aspiration on pre-toilet trained children [6]. 

The results of this systematic review suggest that there are improved healthcare outcomes and utilization when comparing novel urine collection methods to those recommended by national pediatric health organizations and societies. Seven of the studies included in the primary outcome compared first line urine collection techniques to novel methods and reported improved health outcomes using novel methods [5–7, 15–18]. The studies reported that urine bags and clean catch urine collection was slower and less cost-effective than novel methods such as the Quick-Wee method or bladder stimulation technique, and to invasive methods including catheterization and suprapubic aspiration. Only one study compared a non-invasive method (bladder stimulation) to an invasive method (catheterization), and while catheterization had a statistically significantly higher success rate and voiding time, caregivers and heath care providers indicated they were more satisfied with the non-invasive method [6]. The findings suggest that incorporating new evidence and skills such as ultrasound guided catheterization into existing guidelines can improve both the success rate of urine collection and improve patient outcomes [5–7, 15–18].

Finally, our review investigated healthcare provider satisfaction with urine collection methods. Three studies compared novel techniques and methods to first line recommendations. Clinical satisfaction was higher with the novel method compared to the current practice. Kaufman et al. collected clinical satisfaction using a Likert scale but did not explore reasons for preferring the Quick-Wee method over clean catch urine [5]. Baumann et al. and Ravichandran et al. explored a number of reasons why health care providers preferred their chosen methods, including aspects of the procedure itself as well as patient-related factors [6, 14]. 

Overall, there are several strengths and limitations to this study. First, the research team included an experienced health science librarian who developed the search strategy. This resulted in a rigorous search process. Limitations of the study included that there were relatively few studies identified during the systematic review despite urine collection being a common procedure in young children. Of those studies those identified, there was a wide range of outcomes. This meant that we were unable to perform a meta-analysis and investigate the strength of association within these studies, limiting out ability to draw strong conclusions about current urine collection method practices.

Future research is needed to continue to explore urine collection methods for pre-toilet trained children presenting to the EDs with signs and symptoms of a UTI. This review identified that there was limited research into the actual practice variations among clinicians. Future work is needed to determine if physicians adhere to the guidelines recommended by national pediatric societies and organizations. The novel methods identified have the possibility to help inform clinical practice guidelines and allow for improved outcomes in collecting sterile urine samples. However, randomized controlled trials are needed to confirm or refute if the identified novel urine collection methods are associated with better health care outcomes.

## Conclusions

This study demonstrates that there is currently significant practice variation in the urine collection methods choice within and between countries. This study highlights the importance of future research needed to better examine practice variation among clinicians and adherence to national organizations and societies guidelines. Novel methods have increasing utility in practice but are not yet integrated into standard of care. The results identify that additional research can help identify methods that have positive clinical and patient outcomes and integrate them into the guidelines of pediatric societies and organizations.

### Electronic supplementary material

Below is the link to the electronic supplementary material.

Supplementary Material 1

## Data Availability

All data generated or analysed during this study are included in this published article. The search strategy generated during the current study are available in Appendix A and all search strategies are available at 10.34990/FK2/IZ3M32.

## Abbreviations

UTI: urinary tract infection
ED: emergency department
NOS: Newcastle-Ottawa scale
NICE: National Institute for Health and Clinical Excellence

## References

[1] Shaikh N, Morone NE, Bost JE, Farrell MH (2008). Prevalence of urinary tract infection in childhood: a meta-analysis. Pediatr Infect Disease J.

[2] Pediatrices AA. of. Practice Parameter: The Diagnosis, Treatment, and Evaluation of the Initial Urinary Tract Infection in Febrile Infants and Young Children. Pediatrics. 1999;103(4). 10.1542/peds.103.4.843 10103321

[3] Etoubleau C, Reveret M, Brouet D, Badier I, Brosset P, Fourcade L (2009). Moving from bag to catheter for urine Collection in Non-toilet-trained Children suspected of having urinary tract infection: a paired comparison of urine cultures. J Pediatr.

[4] Diviney J, Jaswon MS. Urine collection methods and dipstick testing in non-toilet-trained children. Pediatr Nephrol. 2020. 10.1007/s00467-020-04742-w PMC8172492 32918601

[5] Kaufman J, Fitzpatrick P, Tosif S, Hopper SM, Donath SM, Bryant PA et al. Faster clean catch urine collection (Quick-Wee method) from infants: Randomised controlled trial. BMJ (Online). 2017;357. 10.1136/bmj.j1341 PMC6284210 28389435

[6] Ravichandran Y, Parker S, Farooqi A, Delaroche A. Bladder stimulation for clean catch urine Collection Improved parent and provider satisfaction. Pediatr Emerg Care • [Internet]. 2022;38(1). Available from: www.pec-online.com. 10.1097/PEC.0000000000002524 34475366

[7] Akca Caglar A, Tekeli A, Demir Karacan C, Tuygun N (2021). Point-of-care ultrasound-guided Versus Conventional bladder catheterization for urine sampling in children aged 0 to 24 months. Pediatr Emerg Care [Internet].

[8] Excellence NI, for H, C NICE. Urinary tract infection in under 16s: diagnosis and management. Nice [Internet]. 2018;(August 2007):1–27. www.nice.org.uk/guidance/cg54.

[9] Page MJ, McKenzie JE, Bossuyt PM, Boutron I, Hoffmann TC, Mulrow CD et al. The PRISMA 2020 statement: an updated guideline for reporting systematic reviews. BMJ. 2021;372. 10.1136/bmj.n71 PMC8005924 33782057

[10] McGowan J, Sampson M, Salzwedel D, Cogo E, Foerster V, Lefebvre C (2016). PRESS peer review of electronic search strategies: 2015 Guideline Statement. J Clin Epidemiol.

[11] Veritas Health Innovation. Covidence systematic review software. Melbourne, Australia.

[12] Wells G, Shea B, O’Connell D, Peterson J, Welch V, Losos M et al. The Newcastle-Ottawa Scale (NOS) for assessing the quality if nonrandomized studies in meta-analyses [Internet]. 2012. http://www.ohri.ca/programs/clinical_epidemiology/oxford.asp.

[13] Microsoft Corporation. Microsoft Excel [Internet]. 2018 [cited 2023 Aug 24]. Available from: Retrieved from https://office.microsoft.com/excel.

[14] Brigitte M, Baumann K, McCans SA, Stahmer MB, Leonard (2007). Justine Shults, William C. Holmes. Caregiver and Health Care Provider satisfaction with volumetric bladder Ultrasound. Acad Emerg Med.

[15] Eran Kozer E, Rosenbloom MDG, Gila Lavy N, Rosenfeld MG (2006). Pain in infants who are younger than 2 months during Suprapubic aspiration and transurethral bladder catheterization: a Randomized, controlled study. Pediatrics.

[16] Ho IVA, Lee CH, Fry M (2014). A prospective comparative pilot study comparing the urine collection pad with clean catch urine technique in non-toilet-trained children. Int Emerg Nurs.

[17] Jonathan Kaufman AJ, Knight PA, Bryant FE, Babl, Kim Dalziel (2019). Liquid gold: the cost-effectiveness of urine sample collection methods for young precontinent children. Arch Dis Child.

[18] Lennon R, Krishnamohan A, Fitzpatrick L, Gillett M. CATCH IT: The Effect of Bladder Ultrasound in Decreasing the Time to Collect a Clean-Catch Urine Sample in the Nontoilet-Trained Child A Randomized Control Trial [Internet]. 2023. www.pec-online.com. 10.1097/PEC.0000000000002937 37011266

[19] Agency for Healthcare Research and Quality. Clinical Practice Guideline for Urinary Tract Infection in Children. 2009.

[20] National Collaborating Centre for Women’s and Children’s Health. Urinary tract infection in children diagnosis, treatment and long-term management. London; 2007.

[21] NSW Health. Children and Infants with Fever-Acute Management [Internet]. New South Wales; 2004 Dec [cited 2023 Aug 17]. https://www1.health.nsw.gov.au/pds/Pages/doc.aspx?dn=PD2005_388.

[22] McTaggart S, Danchin M, Ditchfield M, Hewitt I, Kausman J, Kennedy S (2015). KHA-CARI guideline: diagnosis and treatment of urinary tract infection in children. Nephrology.

[23] Buntsma D, Stock A, Bevan C, Babl FE. How do clinicians obtain urine samples in young children? Vol. 24, EMA - Emergency Medicine Australasia. 2012. pp. 118–9. 10.1111/j.1742-6723.2011.01518.x 22313572

