# Supplementary material for: Practice variation in urine collection methods among pre-toilet trained children with suspected urinary tract infection: a systematic review
Source: BMC Pediatr. 2024 May 3;24:294. doi: 10.1186/s12887-024-04751-w (PMC11067245; doi:10.1186/s12887-024-04751-w)
Supplement: Supplementary file 1 — Supplementary Material 1 [file 12887_2024_4751_MOESM1_ESM.docx]

**Appendix A: Search Strategies**

**Search Strategies**

EBM Reviews - Cochrane Central Register of Controlled Trials <September 2021>

Embase <1974 to 2021 October 08>

Ovid MEDLINE(R) ALL <1946 to October 08, 2021>

APA PsycInfo <1806 to October Week 1 2021>

EBM Reviews - Cochrane Central Register of Controlled Trials <September 2021>

Embase <1974 to 2021 October 08>

Ovid MEDLINE(R) ALL <1946 to October 08, 2021>

APA PsycInfo <1806 to October Week 1 2021>

1 Urine Specimen Collection/ 17074

2 (urin* adj5 (captur* or collect* or cultur* or sampl* or specimen*)).tw,kf. 204375

3 (bladder* adj3 (aspiration* or catheter* or stimulation* or puncture*)).tw,kf. 10883

4 (clean catch* or clean void*).tw,kf. 1148

5 ((supra pubic* or suprapubic*) adj3 (aspiration* or catheter* or stimulation* or puncture*)).tw,kf. 4120

6 ((urethral or transurethral) adj3 catheter*).tw,kf. 10613

7 quick wee.tw,kf. 35

8 ((mid stream or midstream) adj3 (catch* or collect* or cultur* or sampl* or specimen*)).tw,kf. 2421

9 sterile urin*.tw,kf. 1308

10 bag specimen*.tw,kf. 71

11 ((collect* or urin*) adj3 bag).tw,kf. 2110

12 ((nappy or urin*) adj3 pad*).tw,kf. 1112

13 or/1-12 [Urine Collection] 231722

14 exp Emergency Medicine/ 59159

15 Emergencies/ 84159

16 exp Emergency Medical Services/ 271235

17 Trauma Centers/ 118242

18 Triage/ 81202

19 Emergency Treatment/ 28654

20 Emergency Services, Psychiatric/ 2844

21 Ambulatory Care/ 93953

22 (emergenc* adj5 (care* or centre* or center* or department* or diagnos* or doctor* or health care or healthcare or hospital* or medicine* or nurs* or patient* or physician* or resident* or room* or service* or therap* or treatment* or unit* or ward* or visit*)).mp. 821034

23 (trauma adj5 (care$ or centre$ or center$ or department$ or diagnos$ or doctor$ or health care or healthcare or hospital$ or medicine$ or nurs$ or patient$ or physician$ or resident$ or room$ or service$ or therap$ or treatment$ or unit$ or ward$ or visit$)).mp. 218872

24 ((urgent or ambulatory or outpatient) adj3 (care$ or visit$ or service$)).tw,kf. 137059

25 (emergency or emergencies or trauma).jw. 346800

26 emergency.in. 397279

27 or/14-26 [Emergency Room Concept] 1587895

28 exp child/ or exp infant/ 5469698

29 (Infan* or infancy or newborn* or new-born* or perinat* or neonat* or baby or baby* or babies or toddler* or minors or minors* or boy or boys or girl* or kid or kids or child or child* or children* or schoolchild* or school child* or pediatric* or paediatric* or peadiatric* or prematur* or preterm* or toilet train* or potty train*).tw,kf. 6792188

30 or/28-29 [Child Concept] 8580069

31 13 and 27 and 30 [Urine + Emerg + Child] 2098

32 31 use medall 728

33 urine sampling/ 16549

34 exp external urine collection device/ 461

35 (urin* adj5 (captur* or collect* or cultur* or sampl* or specimen*)).tw,kw. 204653

36 (bladder* adj3 (aspiration* or catheter* or stimulation* or puncture*)).tw,kf. 10883

37 (clean catch* or clean void*).tw,kw. 1147

38 ((supra pubic* or suprapubic*) adj3 (aspiration* or catheter* or stimulation* or puncture*)).tw,kw. 4109

39 ((urethral or transurethral) adj3 catheter*).tw,kw. 10526

40 quick wee.tw,kw. 35

41 ((mid stream or midstream) adj3 (catch* or collect* or cultur* or sampl* or specimen*)).tw,kw. 2418

42 sterile urin*.tw,kw. 1308

43 bag specimen*.tw,kw. 71

44 ((collect* or urin*) adj3 bag).tw,kw. 2097

45 ((nappy or urin*) adj3 pad*).tw,kw. 1159

46 or/33-45 [Urine Collection] 232145

47 exp emergency health service/ 271235

48 exp emergency medicine/ 59159

49 exp emergency treatment/ 413832

50 exp emergency/ 97378

51 exp emergency ward/ 260806

52 ambulatory care/ 93953

53 (emergenc* adj2 (care* or centre* or center* or department* or diagnos* or doctor* or health care or healthcare or hospital* or medicine* or nurs* or patient* or physician* or resident* or room* or service* or therap* or treatment* or unit* or ward* or visit*)).tw,kf. 525846

54 (trauma adj5 (care* or centre* or center* or department* or diagnos* or doctor* or health care or healthcare or hospital* or medicine* or nurs* or patient* or physician* or resident* or room* or service* or therap* or treatment* or unit* or ward* or visit*)).mp. 218872

55 ((urgent or ambulatory or outpatient) adj3 (care* or visit* or service*)).tw,kw. 135540

56 (emergency or emergencies or trauma).jw. 346800

57 emergency.in. 397279

58 or/47-57 [Emergency Room Concept] 1785744

59 child/ or exp infant/ or exp preschool child/ or exp toddler/ 5346916

60 (Infan* or infancy or newborn* or new-born* or perinat* or neonat* or baby or baby* or babies or toddler* or minors or minors* or boy or boys or girl* or kid or kids or child or child* or children* or schoolchild* or school child* or pediatric* or paediatric* or peadiatric* or prematur* or preterm* or toilet train* or potty train*).tw,kw. 6786115

61 or/59-60 [Child Concept] 8533734

62 46 and 58 and 61 2185

63 46 and 58 and 61 [Urine + Emerg + Child] 2185

64 63 use oemezd 1279

65 urine/ or urinalysis/ 254743

66 (urin* adj5 (captur* or collect* or cultur* or sampl* or specimen*)).tw. 204135

67 (bladder* adj3 (aspiration* or catheter* or stimulation* or puncture*)).tw. 10834

68 (clean catch* or clean void*).tw. 1144

69 ((supra pubic* or suprapubic*) adj3 (aspiration* or catheter* or stimulation* or puncture*)).tw. 4088

70 ((urethral or transurethral) adj3 catheter*).tw. 10494

71 quick wee.tw. 35

72 ((mid stream or midstream) adj3 (catch* or collect* or cultur* or sampl* or specimen*)).tw. 2418

73 sterile urin*.tw. 1308

74 bag specimen*.tw. 71

75 ((collect* or urin*) adj3 bag).tw. 2096

76 ((nappy or urin*) adj3 pad*).tw. 1112

77 or/65-76 [Urine Collection] 414663

78 emergency medicine/ 57881

79 emergency services/ 9188

80 outpatient treatment/ 7134

81 (emergenc* adj5 (care* or centre* or center* or department* or diagnos* or doctor* or health care or healthcare or hospital* or medicine* or nurs* or patient* or physician* or resident* or room* or service* or therap* or treatment* or unit* or ward* or visit*)).mp. 821034

82 (trauma adj5 (care* or centre* or center* or department* or diagnos* or doctor* or health care or healthcare or hospital* or medicine* or nurs* or patient* or physician* or resident* or room* or service* or therap* or treatment* or unit* or ward* or visit*)).mp. 218872

83 ((urgent or ambulatory or outpatient) adj3 (care* or visit* or service*)).tw. 133908

84 (emergency or emergencies or trauma).jw. 346800

85 emergency.in. 397279

86 or/77-85 1869067

87 (Infan* or infancy or newborn* or new-born* or perinat* or neonat* or baby or baby* or babies or toddler* or minors or minors* or boy or boys or girl* or kid or kids or child or child* or children* or schoolchild* or school child* or pediatric* or paediatric* or peadiatric* or prematur* or preterm* or toilet train* or potty train*).tw. [Child Concept] 6720215

88 77 and 86 and 87 [Urine + Emerg + Child] 54341

89 88 use psyc3,psyc4,psyc5,psyc6,psyc7,psyc8,psyc9,psyc10,psyc11,psyc12,psyc13,psyc14,psyc15,psyc16,psyc17 468

90 Urine Specimen Collection/ 17074

91 (urin* adj5 (captur* or collect* or cultur* or sampl* or specimen*)).tw,kw. 204653

92 (bladder* adj3 (aspiration* or catheter* or stimulation* or puncture*)).tw,kw. 11276

93 (clean catch* or clean void*).tw,kw. 1147

94 ((supra pubic* or suprapubic*) adj3 (aspiration* or catheter* or stimulation* or puncture*)).tw,kw. 4109

95 ((urethral or transurethral) adj3 catheter*).tw,kw. 10526

96 quick wee.tw,kw. 35

97 ((mid stream or midstream) adj3 (catch* or collect* or cultur* or sampl* or specimen*)).tw,kw. 2418

98 sterile urin*.tw,kw. 1308

99 bag specimen*.tw,kw. 71

100 ((collect* or urin*) adj3 bag).tw,kw. 2097

101 ((nappy or urin*) adj3 pad*).tw,kw. 1159

102 or/90-101 [Urine Collection] 232273

103 exp Emergency Medicine/ 59159

104 Emergencies/ 84159

105 exp Emergency Medical Services/ 271235

106 Trauma Centers/ 118242

107 Triage/ 81202

108 Emergency Treatment/ 28654

109 Emergency Services, Psychiatric/ 2844

110 Ambulatory Care/ 93953

111 (emergenc* adj5 (care* or centre* or center* or department* or diagnos* or doctor* or health care or healthcare or hospital* or medicine* or nurs* or patient* or physician* or resident* or room* or service* or therap* or treatment* or unit* or ward* or visit*)).mp. 821034

112 (trauma adj5 (care$ or centre$ or center$ or department$ or diagnos$ or doctor$ or health care or healthcare or hospital$ or medicine$ or nurs$ or patient$ or physician$ or resident$ or room$ or service$ or therap$ or treatment$ or unit$ or ward$ or visit$)).mp. 218872

113 ((urgent or ambulatory or outpatient) adj3 (care$ or visit$ or service$)).tw,kw. 135540

114 (emergency or emergencies or trauma).jw. 346800

115 emergency.in. 397279

116 or/103-115 [Emergency Room Concept] 1587322

117 exp child/ or exp infant/ 5469698

118 (Infan* or infancy or newborn* or new-born* or perinat* or neonat* or baby or baby* or babies or toddler* or minors or minors* or boy or boys or girl* or kid or kids or child or child* or children* or schoolchild* or school child* or pediatric* or paediatric* or peadiatric* or prematur* or preterm* or toilet train* or potty train*).tw,kw. 6786115

119 or/117-118 [Child Concept] 8583921

120 102 and 116 and 119 [Urine + Emerg + Child] 2098

121 120 use cctr 137

122 32 or 64 or 89 or 121 2612

123 remove duplicates from 122 1938

124 limit 123 to yr="2000 -Current" 1698

**CINAHL**

| **#** | **Query** | **Limiters/Expanders** | **Last Run Via** | **Results** |
| --- | --- | --- | --- | --- |
| S30 | S13 AND S28 AND S29 | Limiters - Published Date: 20000101-20211231  Expanders - search within the full text of the articles  Search modes - Boolean/Phrase | Interface - EBSCOhost Research Databases  Search Screen - Advanced Search  Database - CINAHL with Full Text | 687 |
| S29 | (MH "Child+") | Expanders - search within the full text of the articles  Search modes - Boolean/Phrase | Interface - EBSCOhost Research Databases  Search Screen - Advanced Search  Database - CINAHL with Full Text | 703,713 |
| S28 | S14 OR S15 OR S16 OR S17 OR S18 OR S19 OR S20 OR S21 OR S22 OR S23 OR S24 OR S25 OR S26 OR S27 | Expanders - search within the full text of the articles  Search modes - Boolean/Phrase | Interface - EBSCOhost Research Databases  Search Screen - Advanced Search  Database - CINAHL with Full Text | 686,806 |
| S27 | AF emergency | Expanders - search within the full text of the articles  Search modes - Boolean/Phrase | Interface - EBSCOhost Research Databases  Search Screen - Advanced Search  Database - CINAHL with Full Text | 88,432 |
| S26 | SO emergency or emergencies or trauma | Expanders - search within the full text of the articles  Search modes - Boolean/Phrase | Interface - EBSCOhost Research Databases  Search Screen - Advanced Search  Database - CINAHL with Full Text | 549,306 |
| S25 | (urgent or ambulatory or outpatient) N3 (care* or visit* or service*) | Expanders - search within the full text of the articles  Search modes - Boolean/Phrase | Interface - EBSCOhost Research Databases  Search Screen - Advanced Search  Database - CINAHL with Full Text | 83,914 |
| S24 | trauma* N5 (care* or centre* or center* or department* or diagnos* or doctor* or "health care" or healthcare or hospital* or medicine* or nurs* or patient* or physician* or resident* or room* or service* or therap* or treatment* or unit* or ward* or visit*) | Expanders - search within the full text of the articles  Search modes - Boolean/Phrase | Interface - EBSCOhost Research Databases  Search Screen - Advanced Search  Database - CINAHL with Full Text | 131,289 |
| S23 | emergenc* N5 (care* or centre* or center* or department* or diagnos* or doctor* or "health care" or healthcare or hospital* or medicine* or nurs* or patient* or physician* or resident* or room* or service* or therap* or treatment* or unit* or ward* or visit*) | Expanders - search within the full text of the articles  Search modes - Boolean/Phrase | Interface - EBSCOhost Research Databases  Search Screen - Advanced Search  Database - CINAHL with Full Text | 348,991 |
| S22 | (MH "Ambulatory Care Facilities+") | Expanders - search within the full text of the articles  Search modes - Boolean/Phrase | Interface - EBSCOhost Research Databases  Search Screen - Advanced Search  Database - CINAHL with Full Text | 16,557 |
| S21 | (MH "Ambulatory Care") | Expanders - search within the full text of the articles  Search modes - Boolean/Phrase | Interface - EBSCOhost Research Databases  Search Screen - Advanced Search  Database - CINAHL with Full Text | 12,729 |
| S20 | (MH "Emergency Services, Psychiatric") OR (MH "Psychiatric Emergencies") | Expanders - search within the full text of the articles  Search modes - Boolean/Phrase | Interface - EBSCOhost Research Databases  Search Screen - Advanced Search  Database - CINAHL with Full Text | 1,502 |
| S19 | (MH "Emergency Treatment+") | Expanders - search within the full text of the articles  Search modes - Boolean/Phrase | Interface - EBSCOhost Research Databases  Search Screen - Advanced Search  Database - CINAHL with Full Text | 61,160 |
| S18 | (MH "Triage") | Expanders - search within the full text of the articles  Search modes - Boolean/Phrase | Interface - EBSCOhost Research Databases  Search Screen - Advanced Search  Database - CINAHL with Full Text | 10,424 |
| S17 | (MH "Education, Emergency Medical Services") | Expanders - search within the full text of the articles  Search modes - Boolean/Phrase | Interface - EBSCOhost Research Databases  Search Screen - Advanced Search  Database - CINAHL with Full Text | 1,636 |
| S16 | (MH "Emergencies") | Expanders - search within the full text of the articles  Search modes - Boolean/Phrase | Interface - EBSCOhost Research Databases  Search Screen - Advanced Search  Database - CINAHL with Full Text | 10,197 |
| S15 | (MH "Emergency Medicine") | Expanders - search within the full text of the articles  Search modes - Boolean/Phrase | Interface - EBSCOhost Research Databases  Search Screen - Advanced Search  Database - CINAHL with Full Text | 12,820 |
| S14 | (MH "Emergency Service+") | Expanders - search within the full text of the articles  Search modes - Boolean/Phrase | Interface - EBSCOhost Research Databases  Search Screen - Advanced Search  Database - CINAHL with Full Text | 65,048 |
| S13 | S1 OR S2 OR S3 OR S4 OR S5 OR S6 OR S7 OR S8 OR S9 OR S10 OR S11 OR S12 | Expanders - Apply equivalent subjects  Search modes - Find all my search terms | Interface - EBSCOhost Research Databases  Search Screen - Advanced Search  Database - CINAHL with Full Text | 13,837 |
| S12 | ((nappy or urin*) N3 pad*) | Expanders - search within the full text of the articles  Search modes - Boolean/Phrase | Interface - EBSCOhost Research Databases  Search Screen - Advanced Search  Database - CINAHL with Full Text | 422 |
| S11 | ((collect* or urin*) N3 bag) | Expanders - search within the full text of the articles  Search modes - Boolean/Phrase | Interface - EBSCOhost Research Databases  Search Screen - Advanced Search  Database - CINAHL with Full Text | 1,444 |
| S10 | "bag specimen*" | Expanders - search within the full text of the articles  Search modes - Boolean/Phrase | Interface - EBSCOhost Research Databases  Search Screen - Advanced Search  Database - CINAHL with Full Text | 24 |
| S9 | "sterile urin*" | Expanders - search within the full text of the articles  Search modes - Boolean/Phrase | Interface - EBSCOhost Research Databases  Search Screen - Advanced Search  Database - CINAHL with Full Text | 131 |
| S8 | (("mid stream" or midstream) N3 (catch* or collect* or cultur* or sampl* or specimen*)) | Expanders - search within the full text of the articles  Search modes - Boolean/Phrase | Interface - EBSCOhost Research Databases  Search Screen - Advanced Search  Database - CINAHL with Full Text | 449 |
| S7 | "quick wee" | Expanders - search within the full text of the articles  Search modes - Boolean/Phrase | Interface - EBSCOhost Research Databases  Search Screen - Advanced Search  Database - CINAHL with Full Text | 7 |
| S6 | ((urethral or transurethral) N3 catheter*) | Expanders - search within the full text of the articles  Search modes - Boolean/Phrase | Interface - EBSCOhost Research Databases  Search Screen - Advanced Search  Database - CINAHL with Full Text | 1,698 |
| S5 | ((supra pubic* or suprapubic*) N3 (aspiration* or catheter* or stimulation* or puncture*)) | Expanders - search within the full text of the articles  Search modes - Boolean/Phrase | Interface - EBSCOhost Research Databases  Search Screen - Advanced Search  Database - CINAHL with Full Text | 922 |
| S4 | ("clean catch*" or "clean void*") | Expanders - search within the full text of the articles  Search modes - Boolean/Phrase | Interface - EBSCOhost Research Databases  Search Screen - Advanced Search  Database - CINAHL with Full Text | 325 |
| S3 | (bladder* N3 (aspiration* or catheter* or stimulation* or puncture*)) | Expanders - search within the full text of the articles  Search modes - Boolean/Phrase | Interface - EBSCOhost Research Databases  Search Screen - Advanced Search  Database - CINAHL with Full Text | 2,421 |
| S2 | (urin* N5 (captur* or collect* or cultur* or sampl* or specimen*)) | Expanders - search within the full text of the articles  Search modes - Boolean/Phrase | Interface - EBSCOhost Research Databases  Search Screen - Advanced Search  Database - CINAHL with Full Text | 18,858 |
| S1 | (MH "Urine Specimen Collection+") | Expanders - search within the full text of the articles  Search modes - Boolean/Phrase | Interface - EBSCOhost Research Databases  Search Screen - Advanced Search  Database - CINAHL with Full Text | 536 |
